# Harvard Odontological Society

**Published:** 1891-09

**Authors:** 

**Affiliations:** Harvard Odontological Society


### HARVARD ODONTOLOGICAL SOCIETY.

The regular monthly meeting of the Harvard Odontological Society was held Thursday evening, March 26, 1891, at Young's Hotel, Boston. The President, Dr. J. E. Stanton, in the chair.

Thomas J. Giblin, D.M.D., read the paper for the evening.

(For Dr. Giblin's paper, see page 614.)

*Dr. Briggs.*—Mr. President, I, in common with every one who has started out in practice, was impressed with the possible dangers arising from the use of the tincture of chloride of iron. As Dr. Giblin says, it was pointed out that the presence of the free acid in that preparation was the danger. As a matter of practical experience, I have learned to take the opposite view from that of the writer. My clinical experience has rather led me to feel that the tincture of chloride of iron simply stained, and that it did not, to a great extent, injure the teeth. Of course, as Dr. Giblin has pointed out, if a tooth is put in the tincture it does attack it very freely, but its position in the mouth is very different. If any acid gets into the mouth, the action of the salivary glands is to increase their flow,—it is nature's means of diluting that acid, and is done in so short a time that there seems to be little opportunity to attack the teeth.

I have come to the conclusion that the condition of the teeth in patients using chloride of iron is caused not so much by the medicine as by the systemic condition which called for the medicine. The condition for which the chloride of iron is used is generally a pretty serious one,—it is usually attended with a great deal of wasting. We speak of it as a tonic, but unlike many tonics it is not for the system that is simply "out of tone." In fact, a person is out of tone oftentimes when he is apparently pretty well nourished, when he has plenty of blood, and there is no wasting disease. In that condition the tincture of chloride of iron is not necessary; in fact, it produces a plethora if given at that time. In many cases where it has been given unguardedly it has produced this plethoric condition, and patients will say that they cannot bear iron because it gives them this full feeling. In the cases where it is given, as the writer has pointed out, it is to supply the red corpuscle of the blood, in the wasting conditions of scrofula, phthisis, and other diseases of like grave nature.

I, of course, am speaking without preparation, not knowing the line of thought of the writer until to-night, but should be very glad to hear the impressions of others on this subject. To sum up, the conclusion at which I have arrived, as a result of what experience I have had with this drug, is, that it acts merely to stain the teeth and make them unsightly, but the mischief, when it has been coincident with the taking of the iron, has been due to the condition of the patient rather than the presence of the drug in the mouth. What the nature may be of the secretions after it has entered into the system,—whether secondarily it may be a medicine of bad import to the teeth,—I have no knowledge.

*President Stanton.*—Dr. Giblin has pointed out, to me at least, a certain phase of the question, perhaps not directly bearing upon his paper, but one which I think will surely come home to most of you, and that is, in regard to a thoroughly good understanding between the practice of medicine and the practice of dentistry. It seems to me every day that the dentists and the physicians are going to conflict more and more unless they understand each other better. Within the past year I have had patients from physicians who have sent them to me and indicated a certain line of treatment, insisting on that treatment as physicians. Recognizing that the physician—at present surely—has the better hold, I concede the point and do my best under the circumstances. Of course, very many times it is a failure from a stand-point of dentistry. The patient goes into it with a full understanding of my position in the matter, and the result, easily foretold, is no surprise, and so far as

I know I have been sheltered from any blame in connection with it, but I know it is perfectly possible for us to suffer severely from the influence of physicians, and especially from physicians of the homœopathic school. If they practise true homœopathy, as many do in the city of Boston,—perhaps more here than anywhere else,—the drugs absolutely necessary for our purpose will conflict with their remedies, so that it is pretty hard sometimes to know what line to take.

*Dr. Cooke.*—The president's remarks bring to mind a case of mine which will serve to illustrate the point he speaks of. A patient came to me with a large growth upon the inside of the lower jaw, growing between the teeth. I cut a section from it and had it examined by an expert, and also showed the tumor to him in the mouth. He pronounced it to be of a serious nature, but thought there would be no harm in my trying, for a time at least, to see if I could cure it. I cut it with a bur and lancet, and from time to time cauterized it. In a short time there was but a point remaining. When left to itself for a week or ten days it would grow up again, and I was obliged to renew treatment. The patient consulted a physician for an eruption on the face. He prescribed certain drugs for her to use, and during their interview she told him that this growth on the gum was being cauterized from time to time with nitrate of silver, whereupon the physician said that this treatment must be stopped, as the nitrate of silver would interfere with the remedies which he had prescribed. The treatment therefore stopped. That was about a year and a half ago. I did not see the patient again until a few days ago. The growth was about as large as it was before. Where there is a case in which the services of both dentist and physician are required, the patient will invariably follow the physician's advice.

*Dr. Werner.*—I have seen but little of the injurious effect due to the use of the tincture of chloride of iron spoken of by the essayist. The patients mentioned by him were undoubtedly in a low state of vitality or resisting power.

Dr. Briggs's remark is very forcible, to the effect that the acid is almost immediately neutralized by the increased flow of saliva caused by the presence of acid in the mouth. We are inclined to expectorate or swallow very soon after acids are tasted, on account of the increasing quantity of saliva, while the tooth in the bottle has no alternative but to submit to decalcification.

*Dr. Niles.*—It seems to me very true that a weak and enfeebled condition of the system might affect the vital resisting power of the teeth, but at the same time it will also affect the chemical constituents of the saliva. The normal reaction of the saliva is alkaline, in health, when secreted in abundance, but if a person is ill, the saliva may become insufficient to counteract the effects of an acid ; therefore, if the tinctures be given under these circumstances they have a better chance to act upon the teeth. I have never seen a case, where the tinctures have not been administered, that the teeth have deteriorated in the manner referred to,—unfortunately it has always been where the tinctures have been introduced. I know that the physicians endeavor to show that it is due to the condition of the health and not to the medicines given, but I once had an opportunity to disprove this very decidedly. Some time ago a patient of mine was under treatment by one of the leading physicians of this city,—a man well known and of unquestioned reputation in his profession. He gave this patient lime-juice as a gargle, with instructions to be used every two hours during the day. This treatment had been continued for two weeks, and having seen the patient just before she was ill, and seeing her again just after she recovered, I had an opportunity to mark the result, and found her teeth very much decalcified over the entire surfaces, the enamel being very chalky. I asked her what she had been doing, and on hearing her story I promptly told her that the medicine she had been using had probably acted upon her teeth. The lady was very indignant, as she had asked the doctor, before commencing treatment, if the medicine would injure her teeth, and he said, " Oh, no, you need not be at all alarmed ; if it does, I will get you a new set." She reported to him what her dentist had told her, and he replied that her dentist was an alarmist and an extremist, which she promptly reported to me. I asked her if she had any of the medicine left that she had been using? She said she had, and brought it to me. I divided the medicine into two parts. I put a part of a tooth into each solution, and told her to keep one of the bottles three days and then take it to Dr. ——, and show him the result. The effect of the medicine was identical with that shown upon her teeth. The physician made a great bluster and went around to his dentist, who told him it was all a humbug, and that it would have no effect whatever on the teeth in the mouth. Both these men—the dentist and the physician—are well known to you all. Tincture of chloride of iron is not the only medicine that will affect teeth. There are many other remedies in common use that will produce the same effect if not cautiously given and the danger guarded against by the use of alkalies.

*Dr. Giblin.*—I only wish that my experience had been as happy as that of Dr. Briggs and Dr. Werner in the matter of never witnessing the injurious effect of the tincture of chloride of iron upon the teeth, but since the appointment was made for me to read a paper I have been more attentive to this matter than ever before, and I have had no less than fifteen patients since that time,—a few days over a month,—whom I could assure you had been victims of the tincture of chloride of iron. Only yesterday afternoon a lady came to my office for whom, a year and a half ago, I repaired what damage had been done to her teeth and pronounced them in good condition. When I saw her yesterday I was very much surprised, as some of the molars were disintegrated down to the margin of the gum, and there were numerous approximal cavities in the anterior teeth. I asked her if she had been using medicine, and she told me she had been taking tincture of iron for over a year. There was very little stain upon her teeth,—they were all simply disintegrating. The cases which we particularly notice, and ask if they have been using iron, are those cases where the teeth have been discolored, but those are just the cases in which the least damage is done. You will find that the iron used in those cases has been in the form of an elixir, being generally combined with syrup and alcohol, and on placing a tooth in the solution for twenty-four hours there is no effect, other than to adhere to the tooth and stain it, but if you place that same tooth in a solution of tincture of chloride of iron in water for the same length of time you will find that the enamel is quite easily scraped off with an instrument. The saliva has precisely the same effect as water. In a person who is using the chloride of iron it is never sufficiently alkaline to counteract its effect, and if it is acid when it is used it will have no neutralizing effect, but increase its activity rather than diminish it.

In my paper I said that I would not suggest a remedy, because I thought it not our duty and our privilege, although I think I could if I felt free to do so. I think, in the first place, that there are other salts of iron that will have as good an effect on the system as the tincture of chloride of iron, but the latter is more commonly used because it is a very cheap and effective preparation. The other salts of iron are usually expensive, such as the citrate of iron, and most patients will question a druggist's bill rather than a doctor's. I think there are few who are aware of the extent to which the chloride of iron is used, and the result of my investigations on this subject was a surprise to me.

I do not maintain that the tincture of chloride of iron always has this bad effect, as there are some physicians who insist upon precautions being taken to prevent its action, but I do not think it is a universal practice among physicians to caution patients. This week I saw a prescription which was given to a lady, which read something like this: "Two drachms of tincture chloride of iron to one ounce of water and a certain quantity of syrup,—teaspoonful to be taken three times a day, after meals," with no further directions or cautions. I have no doubt but what the effects of it will be seen in a very short time.

*Dr. Briggs.*—I would like to ask Dr. Giblin if from his experience he would undertake to say positively whether in these cases it is the primary or secondary effect of the chloride of iron, if he can be sure that the effect which he speaks of is the result of the tincture of chloride of iron as taken into the mouth, or the effect on the system after it has gone into the stomach, and if he thinks it would have as serious an effect if the experiment could be tried of giving it to a person in perfect health?

*Dr. Giblin.*—That is a difficult question to answer. In the first place, I have never met a person in my experience who has taken this tincture while in perfect health, but in cases of local lesion, such, for instance, as diphtheritic sore throat, where it is a frequent remedy, its effect is noticeable. Since paying attention to the subject a college friend of mine was troubled with diphtheritic sore throat, and tincture of chloride of iron was prescribed. They used the medicine in dilution, and I noticed in his teeth a great many white spots, and since writing the paper I have noticed that the action of acid on the teeth always begins with a white spot, especially the hydrochloric. If a tooth is put in a solution of hydrochloric acid, the first appearance is a white spot, and then it rapidly covers the whole surface. I could not reach my friend lately, or I would have been able to make some mention of the further progress of his case. I thought it was the free acid that caused these white spots and carried on the work of disintegration. In the works on therapeutics I could find no mention as to the manner in which it affects the teeth.

*Dr. Briggs.*—I do not wish to go any further in questioning the injurious effect of the tincture of chloride of iron as it goes through the mouth into the stomach. My clinical experience with regard to the effect of this medicine has been so limited that I would not undertake to refute Dr. Giblin's careful investigations, but it did not seem to me that it could have this effect in merely passing through the mouth. I would also say that I have never seen acid saliva from the parotid glands. Possibly it does exist, but I have never yet happened to test it at a time when it was acid. Those are the largest glands, and those are the glands which, if an acid be introduced into the mouth, will increase their secretion, while the acid glands will be checked for the time. The effect of an acid in the mouth is to increase the flow of an alkaline gland, and *vice versa;* the effect of an alkali increases the flow of the acid glands, and I supposed on the strength of this known principle that saliva would correct acidity by its alkalinity,—not merely diluting as water would do. The tincture of chloride of iron is effective, and at the same time the cheapest of the iron preparations. You will find these cases more frequently where there are reasons for economy, and I may not have seen them on that account.

*Dr. Reilly.*—I cannot help adding a little testimony in the way of an endorsement to Dr. Giblin's paper. I must admit that I have had a number of cases in a certain class of my patients, and looking at them now, after the remarks that have been made, it strikes me as a little singular that this extreme decay and disintegration of the teeth should be so coincident with the use of tincture of chloride of iron. They have become so frequent that, under certain aspects, I ask patients if they have been using it. I had an opportunity to notice the effects of iron in my own family, though in another form (the syrup of the iodide of iron), and I know positively that it has had an injurious effect upon the teeth of my children; that is, coincident with the use of it the teeth have given out. The chloride, as Dr. Briggs has said, is used for reasons of economy, and it is used to such an extent that I know mothers go to the drug-store and buy it, and keep it in the house as a medicine. I have seen cases of that kind show a destructive effect so marked that there is no question in my mind that it is a most powerful agent in tooth destruction.

*Dr. Hopkins.*—Does Dr. Giblin think that the iron has anything to do with the black discoloration sometimes seen on gold fillings?

*Dr. Giblin.*—I don't know what that discoloration is caused by. I used Steurer's gold when it first appeared, and the discoloration was very perceptible on that. If a filling of Steurer's gold were inserted alongside of a gold-foil filling, the Steurer's gold would be stained while the foil would remain bright. It is maintained that this discoloration is caused by particles of steel dropping from our plugger points during an operation, thus oxidizing the surface of the filling and extending around the edges, but I think that is hardly probable.

I would like to occupy a few moments of your time in drawing your attention to the difference between the syrup and alcohol solution, and the water solution, of tincture of chloride of iron. The oxide that is formed when the syrup and alcohol solution is used is an anhydrous oxide. It is very dark and dense, and when deposited upon the teeth protects them from the free acid until it is removed by the natural events in the mouth. In the water solution, the oxide formed is a purely water oxide. The chloride of iron unites with so many parts of water, and forms a simple oxide of iron. This oxide is flocculent, and does not adhere to the teeth. This can be shown in a test tube.

*Dr. Bigelow.*—Dr. Giblin has intimated that he should not suggest to physicians any means of neutralizing the effect produced by the tincture of chloride of iron, on the ground that the means used might antagonize the systemic effect desired by the physician, and that it was not his duty to do so. If the theories advanced by him are correct, I, as a dentist, should like to hear the practical side of the question and to know what the remedy is. If we are to use our influence with the medical profession in this matter, we should know the remedy, and I would like to hear what it is.

*Dr. Giblin.*—To reiterate what I said in the paper, the reason why I think it not to be proper for us to suggest a remedy for the evil is from the fact that, not being acquainted with the general effects of the medicine through the system, we may not understand the methods of administering. It is maintained that the tincture of chloride of iron in a syrup solution is not so tenable on the stomach as the water solution, and that a weak stomach would reject it. But even if this were not so, it may not be the reason why it is not always prescribed in this form, as some physicians might think that an alcohol solution would produce an appetite for alcohol in the patient who would use it. We know that such appetites are caused and cultivated by the use of medicine. You will all remember some instance where a habit has been formed—the morphia habit especially—in this way. One of the means of neutralizing the effect is an alkaline mouth-wash. Every patient will find at home a little saleratus that will produce the desired effect.

*Dr. Werner.*—I think that among the better class of practitioners the tincture of chloride of iron is but very little used, other preparations of iron being usually employed. I do not wish to combat the good points brought out by the essayist. I am speaking upon this question as I understand it simply. I think that coming in contact with fifteen cases of this kind in six weeks is an extraordinarily large number,—fifty per cent. more than I have seen in fifteen years. I think Dr. Giblin would be doing his duty in speaking to the practitioner who prescribes this drug so indiscriminately.

*Dr. Briggs.*—I have never seen any works on therapeutics where it has been described, nor in the dispensatories, that have not expressly laid down the fact that it was very injurious to the teeth and that caution should be used. Whatever may be our private opinion of what they are in the habit of doing, if they intend to follow the recognized standards of medical practice they cannot do otherwise than to direct that it be used with care, and, if deemed necessary, something should be prescribed to counteract its effect upon the teeth.

*Dr. Clifford.*—Does Dr. Briggs think there is any deleterious action from the stain of the piano-wire?

*Dr. Briggs.*—I have seen the staining of teeth from the piano-wire, and quite serious staining in places where regulating appliances have been left on for a long time, but I have not seen any erosion or wasting of enamel at that point. I imagine it is simply the oxide of iron on the teeth.

*Dr. Niles.*—Some time ago Dr. Hawes, while instructor in the Harvard School, made some experiments and went through a long list of the salts and tinctures kept at the druggist's, and he found that a majority of them were held in solution only by the acidity. We all know that most acids will dissolve tooth-structure; hydrochloric acid, perhaps, has the most powerful effect, inasmuch as the products formed are readily soluble in water. Sulphuric acid does not act as rapidly. Sulphates of calcium are not readily soluble in water, and as there is a long list of tinctures which are only held in solution by rendering them more or less acid, it would be an interesting study for some young man to follow up this line of experiment and find out what salts are injurious to the teeth. I was told by a physician recently that there were no allusions to this matter in the lectures in the schools. It would be perfectly proper to bring it to the attention of the medical instructors, and I think it would be kindly received by them.

H. L. Upham, D.M.D.,
*Editor Harvard Odontological Society.*

